# Add-on Lamotrigine Treatment for Subsyndromal Depression after Manic or Mixed States in Bipolar Disorder Improved the Quality of Life

**DOI:** 10.1155/2012/736521

**Published:** 2012-09-23

**Authors:** Katsumasa Muneoka, Katsushi Kon, Masaharu Kawabe, Rui Ui, Taichi Miura, Touta Iimura, Shou Kimura

**Affiliations:** ^1^Kimura Hospital, 6-19 Higashi-Honcho, Chuo-Ku, Chiba, Chiba-shi 260-0004, Japan; ^2^Department of Psychiatry, Graduate School of Medicine, Chiba University, Chiba 260-8670, Japan

## Abstract

Two cases of patients experienced subsyndromal depression after manic or mixed hypomanic and depressive episodes due to bipolar I (case 1) and II (case 2) disorders prior to the use of lamotrigine. Case 1 showed episodes of mood switching induced by antidepressants and seasonal mood instability. Case 2 showed hippocampal atrophy and a persistent dull headache that preceded the use of lamotrigine. Both were successfully treated with add-on lamotrigine therapy, and the dull headache was effectively treated with olanzapine. Both patients improved in social activity and work performance after these add-on treatments. Thus, add-on treatment with lamotrigine alone or in combination with olanzapine was an effective strategy to improve the quality of life in bipolar depression. Subsyndromal depression that present after the disappearance of the manic or mixed state was suggested to be practical indication for the use of lamotrigine.

## 1. Introduction

Recent reports have demonstrated that, in bipolar I and II disorders, patients spent over threefold more time in depression than in manic or hypomanic symptoms [1–3]. Depression was reported to affect the quality of life (QOL) or subjective life satisfaction in patients with bipolar disorders [4–6]. Therefore, depressive states in bipolar disorders are a primary reason for functional difficulties and social impairment. 

It has been proposed that bipolar depression warrants pharmacotherapy distinct from that for unipolar depression. Antidepressants have limited effectiveness [7, 8], and they run the risk of inducing mania [9, 10]. Although acute mania could be effectively treated with typical mood stabilizers, including lithium, valproate, or carbamazepine, or with first- and second-generation antipsychotics, these medications do not seem to be always suitable for treating bipolar depression [11]. However, both quetiapine and olanzapine are second-generation antipsychotic agents and both have been shown to be accompanied by efficacy in the depressed phase of bipolar disorder, quetiapine in both bipolar I and II [12] and olanzapine in only bipolar I [13].

Lamotrigine is a recently approved mood stabilizer that effectively prevented relapses in patients with bipolar I disorder with recent depressive episodes [14, 15]. Additionally, lamotrigine was an effective maintenance therapy for patients with a predominant depressive polarity in bipolar disorder [16]. Lamotrigine was also reported to be an effective add-on therapy [17]. Furthermore, lamotrigine showed mood-stabilizing potential, because it suppressed weekly mood fluctuations [18]. On the other hand, lamotrigine did not appear to elevate the risk of switching the mood to a manic state [19]. Thus, lamotrigine had the potential to prevent relapse and maintain recovery from depression in bipolar disorders, without contributing to the risk of mood switching.

However, practical indication for the monotherapy of lamotrigine in acute bipolar depression seems to be controversial [20, 21]. An independent meta-analysis suggests a beneficial effect on depressive symptoms in the depressed phase of bipolar depression, especially in patients show severer scores for baseline depression [22]. This study presents evidence of lamotrigine efficacy for treating sub-syndromal depression that appeared after manic or mixed hypomanic and depressive episodes and impairing social activity in patients with bipolar I or II disorder.

## 2. Case History

### 2.1. Case 1

A 42-year-old male high school teacher, started treatment for depression at a clinic in 1997 at 29 years old. For ten years, he experienced several episodes of depression and recovery. He underwent seasonal mood fluctuations, with a trend of euphoria in spring and summer and depression at the beginning of autumn. In August 2007, he became extremely talkative, hyperactive, irritable, and he began to squander money. He was diagnosed with bipolar disorder I according to the Diagnostic and Statistical Manual of Mental Disorders (DSM)-IV-TR. He was suspended from his job and hospitalized. He responded to pharmacotherapy with lithium, valproate, and risperidone, and was discharged in December 2007. After the discharge, he fell into a depression. He complained that it was annoying to do anything, particularly in the mornings. He received antidepressant treatments with milnacipran at the maximal dose of 100 mg/day for three months or 10 mg/day mianserin for 2 weeks, but neither treatment was effective. He could not return to his job due to depression. Next, he was treated with maprotiline at a daily dose of 20 mg; four days after starting this treatment, he abruptly experienced manic symptoms, including talkativeness, irritability, money squandering, sleeplessness, and a feeling of intense excitement. In August 2008, he was hospitalized again. Upon evaluation, he received a score of 136 on the CLINICAL GLOBAL IMPRESSION-BP Version (CGI-BP), and he was considered severely ill according to the overall bipolar illness on the Young Mania Rating Scale (YMRS). In October 2008, after undergoing pharmacotherapy, these evaluations were reduced to 4 and minimally ill, respectively. At that time, he was discharged with a prescription of 1000 mg/day lithium, 1200 mg/day valproate, and 3 mg/day clonazepam. However, his depression returned.

**Figure 1:**
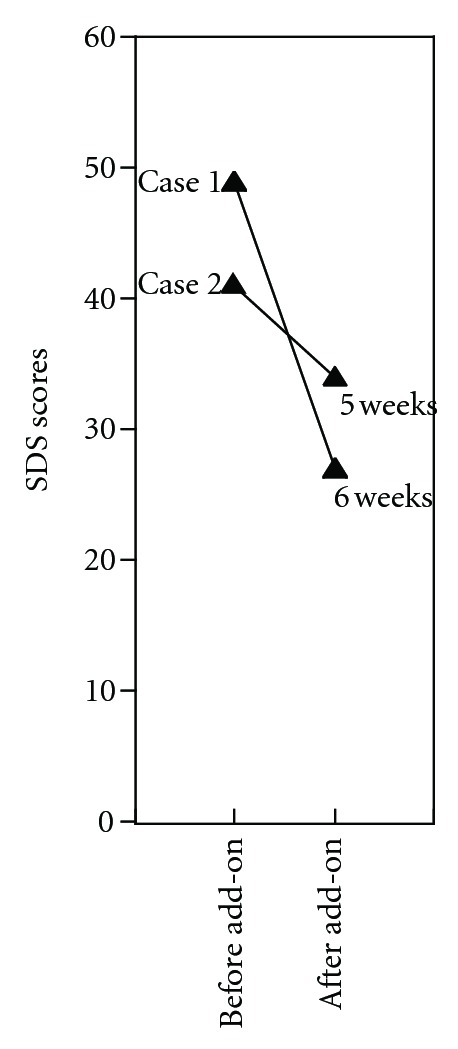
SDS scores before and after the add-on treatment with lamotrigine. SDS evaluations were performed before, and at 5 and 6 weeks after beginning lamotrigine.

In January 2009, he began treatment with lamotrigine at a daily dose of 50 mg. The lamotrigine dose was elevated gradually to 100 mg/day; the dose of lithium was reduced to 400 mg/day; the clonazepam was discontinued. After one month of treatment with lamotrigine, his depression was alleviated. His score declined on the Zung Self-Rated Depression Scale (SDS; Figure 1). In April 2009, he began a rehabilitation program in his workplace. In October, the lamotrigine dose was increased to 150 mg/day when his depression worsened, and this treatment was effective at preventing further episodes. During the rehabilitation program, no apparent depressive moods or manic states recurred. He returned to his job full time in March, 2011.

### 2.2. Case 2

**Figure 2:**
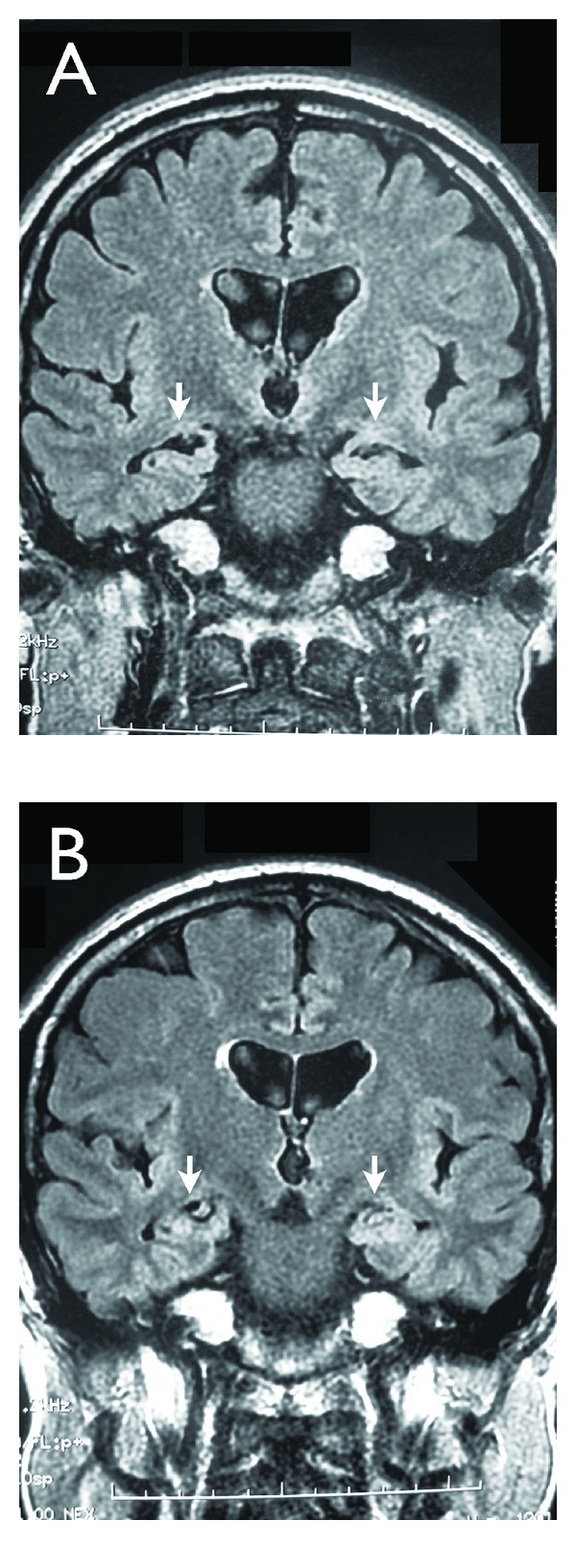
Fluid-attenuated inversion recovery MRI images of the brain in Case 2. MRIs were performed (A) in December 2008 and (B) November 2011. Arrows indicate the hippocampus.

A 47-year-old male pharmaceutical advertiser had experienced depression, anxiety, and insomnia, shortly after his transfer to a different branch in May 2006 at 46 years old. He was suspended from his job and received pharmacological treatment in a clinic. In October 2006, he attempted to return to his job, but he failed due to irritability and aggressiveness. During that period, he had difficulty suppressing the urge to declaim people on the street when they displayed bad social manners. Due to depressive symptoms, anxiety, and irritability, he entered a hospital in November, 2008. He was diagnosed with bipolar disorder II according to the DSM-IV-TR. In December 2008, brain magnetic resonance imaging (MRI) indicated bilateral hippocampal atrophy (Figure 2(A)). He was treated with mood stabilizers, including 600 mg/day lithium and 600 mg/day valproate in combination with 30 mg/day amitriptyline. Trihexyphenidyl was also administered to calm a mild tremor in the hands and trunk of the body, which intensified with tension. During the hospitalization, he received a CGI-BP score of 18, and he was considered moderately ill according to the Overall Bipolar Illness of YMRS. He was discharged in February 2009 with an SDS score of 34, and his CGI-BP and Overall Bipolar Illness of YMRS evaluations were 0 and Normal, respectively.

After the discharge, he complained of depression, dry mouth, and a dull headache in the forehead. In March 2009, the SDS score deteriorated to 41. An add-on treatment of lamotrigine was begun at 25 mg every other day, and this was increased gradually to 100 mg/day. Conversely, amitriptyline and lithium were tapered down and then ceased. After 5 weeks of the add-on treatment, his depression ameliorated, and the SDS score had reduced to 34 (Figure 1). In addition, the dry mouth and hand tremors had ameliorated. He began a rehabilitation program at the workplace in September, 2009. However, the dull headache continued and intensified when he was tired. In August 2010, olanzapine was added and the patient responded well. In October 2010, he returned to work. In November 2011, MRI analysis showed beneficial changes in the hippocampus (Figure 2(B)).

## 3. Discussion

The present case studies showed that bipolar depression was successfully treated with add-on lamotrigine therapy. Both patients experienced subjective depressive feelings that emerged after a remission from manic or mixed hypomanic and depressive states. Add-on lamotrigine therapy alleviated these symptoms, reduced the SDS scores, and did not cause mood switching to manic or hypomanic states. Both patients recovered normal social activity and returned to their jobs after a period of rehabilitation. Of note, alleviation of subjective depression is critical for improving the QOL.

Case 1 exhibited a high risk of mood switching induced by antidepressants. In addition, seasonal mood instability was noted, a pattern proposed to be characteristic of patients with bipolarity [23].

In case 2, add-on lamotrigine treated depression and anxiety-related tremors. Moreover, the dull headache responded to olanzapine treatment. Olanzapine has been reported to be efficacious for treating comorbid anxiety symptoms in patients with bipolar disorders [24, 25]. It was suggested that olanzapine had neuroprotective or neurotrophic properties [26]. The response of this patient suggested that the treatment ameliorated hippocampal atrophy. These findings were consistent with previous findings [27, 28]. However, although the neurotrophic effects of olanzapine might be relevant to the recovery, mood stabilizers also have neurotrophic effects [29].

The present cases described patients with jobs that required high intellectual performance. Therefore, these bipolar disorders impacted job performance due to the effects on neurocognitive functions, including impairments in executive function, verbal learning, and processing speed [30–32]. We speculate that the improvement in subjective depression may have contributed to neurocognitive recovery. In the argument whether pharmacotherapy affects neurocognitive function [33, 34], lamotrigine was suggested to confer a favorable neurocognitive profile [35].

In conclusion, the present case studies demonstrated that add-on lamotrigine improved subsyndromal depressive symptoms and improved the QOL in bipolar I and II disorders. Thus, subsyndromal depression may be practical indication for the use of lamotrigine.
